# Supplementary material for: Aberrant epigenome in iPSC‐derived dopaminergic neurons from Parkinson's disease patients
Source: EMBO Mol Med. 2015 Oct 29;7(12):1529–46. doi: 10.15252/emmm.201505439 (PMC4693505; doi:10.15252/emmm.201505439)
Supplement: Supplementary file 1 — Expanded View Figures PDF [file EMMM-7-1529-s001.pdf]

Expanded View Figures

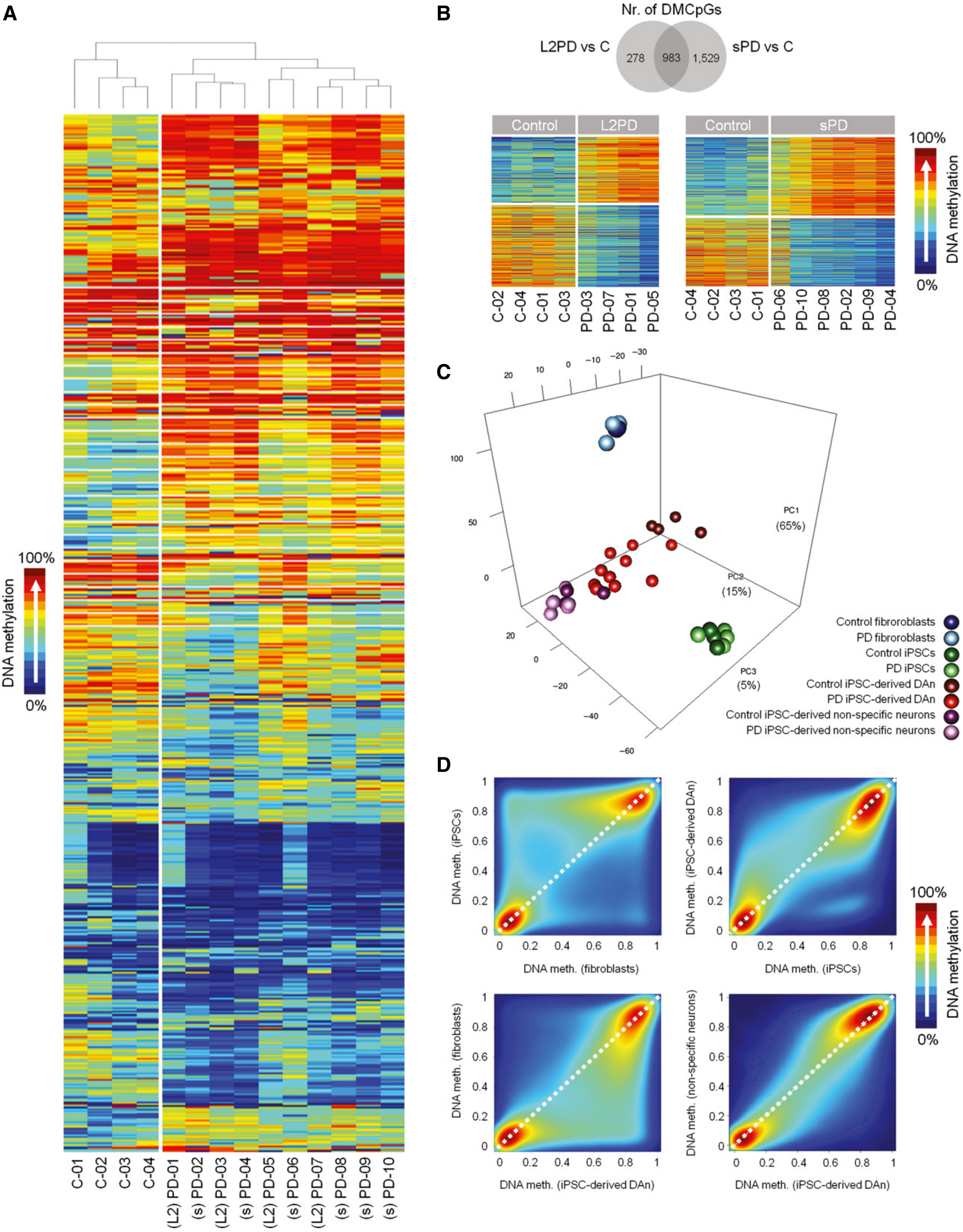

Figure EV1.

© 2015 The Authors

Figure EV1. Identification of epigenetic changes associated with monogenic and sporadic PD in iPSC-derived DAn but not in other cell types.

A Unsupervised hierarchical cluster analysis of methylation data from 28,363 CpG sites with variable methylation values (SD > 0.1). This analysis shows different DNA methylation profiles in PD patients as compared to controls.

B Venn diagram representing the number of differentially methylated CpGs (DMCpGs) in iPSC-derived DAn from monogenic (L2PD) or sporadic PD (sPD) patients with respect to controls (n = 14). Most DMCpGs in L2PD were common to sPD (78%) (upper part of the panel). Heatmaps of DMCpGs detected between L2PD and controls (n = 1,261) and between sPD and controls (n = 2,512) (lower part of the panel). This analysis shows that the methylation patterns of L2PD and sPD, either pooled as shown in Fig 2D, or as independent groups, are different from controls (Wilcoxon rank test for independent samples (n = 14) with delta-beta above |0.25| and FDR-adjusted $P < 0.05$). C, control.

C Principal component analysis (PCA) of methylation data from the 28,363 CpG sites with variable methylation values (SD > 0.1) in fibroblasts, undifferentiated iPSC, iPSC-derived DAn, and iPSC-derived neurons (not-DAn). The PCA demonstrates that sample variability is attributable, in a decreasing manner, to (i) the cell type as expectable, (ii) the condition PD or control only in iPSC-derived DAn, and (iii) the inter-individual variability in a much lesser extent. These results indicate that methylation differences identified in PD were specific for DAn.

D DNA methylation analysis across all studied cell types as illustrated in one representative control (C-02). Density scatter plots of CpG-wise DNA methylation level differences, and density color code, for mean DNA methylation data of all autosomes comparing between fibroblasts and undifferentiated iPSCs, between undifferentiated iPSCs and iPSC-derived DAn, between iPSC-derived DAn and fibroblasts, and between iPSC-derived DAn and iPSC-derived neurons (not-DAn). This analysis shows that the methylome is modulated during the *in vitro* cell reprogramming and differentiation processes.

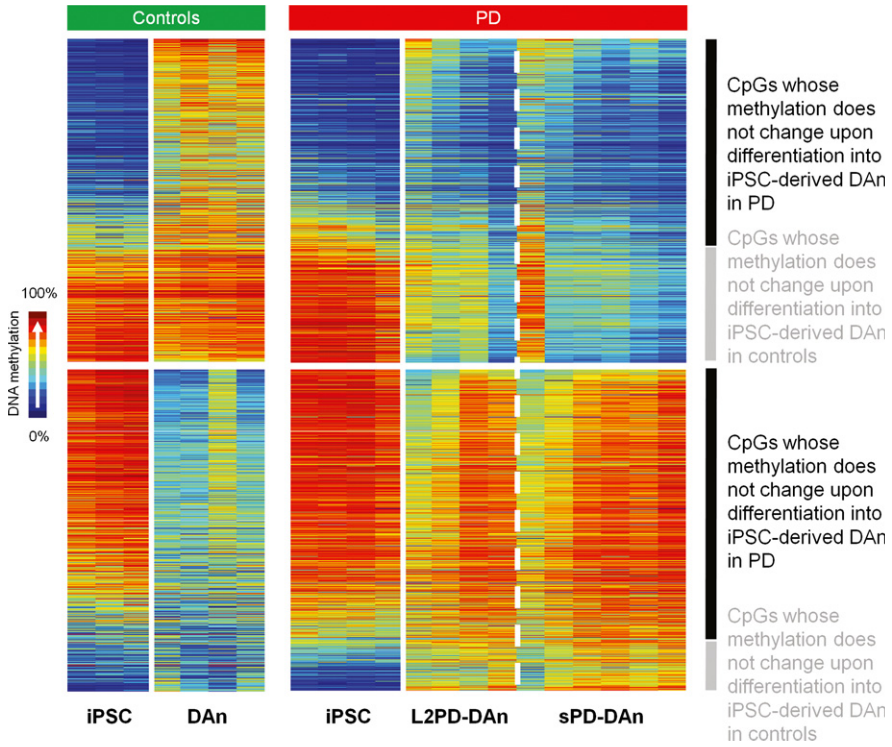

Figure EV2. Differentially methylated CpGs (DMCpGs) detected in PD iPSC-derived DAn (n = 2,087) and comparison of their methylation status with respect to undifferentiated iPSCs.
DNA methylation data derived from Fig 2D reorganized to show that a total of 75% of the 2,087 DMCpGs did not change in the differentiation from iPSCs to DAn in PD (right), whereas only 40% DMCpGs remained unchanged in controls (left), suggesting an incomplete epigenomic remodeling in PD in spite of the successful reprogramming and differentiation into mature DAn (Wilcoxon rank test for independent samples (n = 14) with delta-beta above |0.25| and FDR-adjusted $P < 0.05$).

© 2015 The Authors

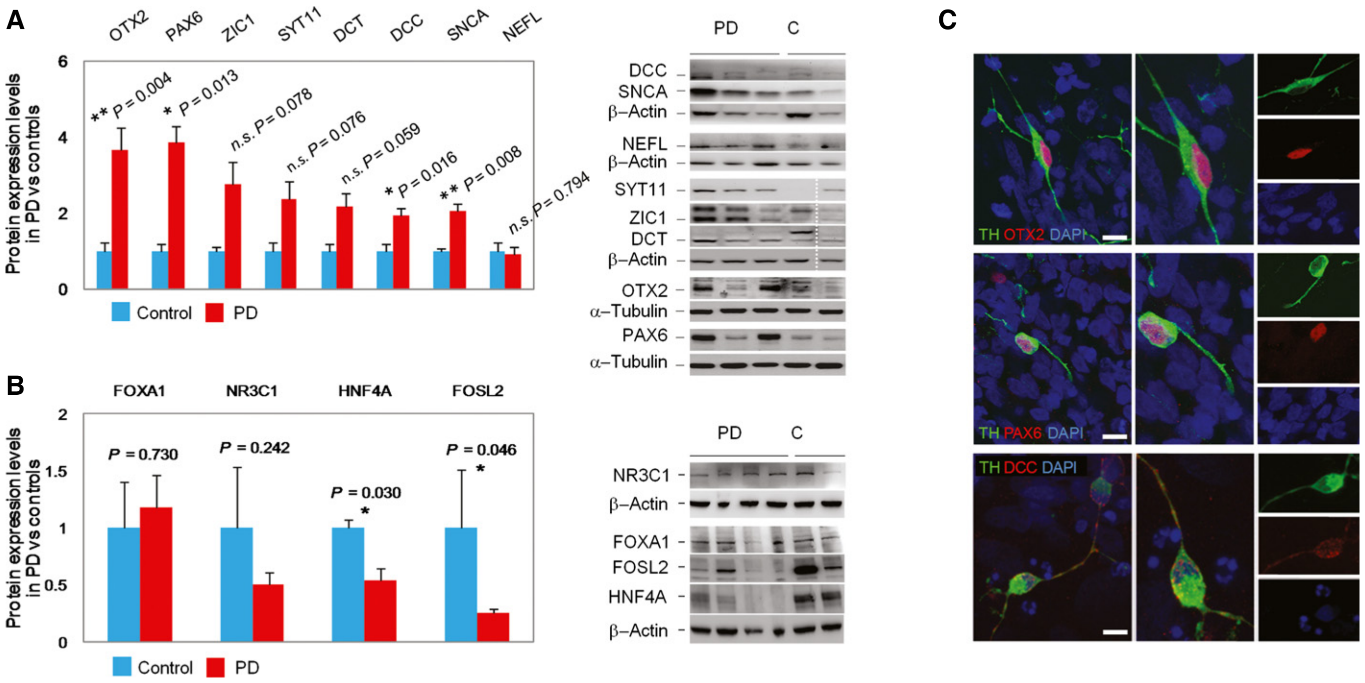

Figure EV3. Identification of protein expression deregulation in PD iPSC-derived DAn.

A Protein levels of top upregulated differentially expressed genes (DEGs) in PD iPSC-derived DAn as compared to controls (n = 14). Band densitometric quantification analysis of protein levels as determined by immunoblot and normalized to the expression of b-actin or a-tubulin. Two-tailed Student's t-test (**$P < 0.01$, *$P < 0.05$). All samples were studied in at least three independent experiments. Data are represented as group mean ± SEM. This analysis shows protein expression upregulation for all DEGs except NEFL (right). Representative immunoblots from PD patients (PD) and controls (C). Discontinuous white line indicates samples from the same blot re-grouped for representation (left). See also source for Fig 7.

B Protein levels from key transcription factors (TFs) showing reduced gene expression and association with enhancer hypermethylation in PD iPSC-derived DAn as compared to controls (n = 9). Band densitometric quantification analysis of protein levels from key TFs normalized to the expression of b-actin. Two-tailed Student's t-test (**$P < 0.01$, *$P < 0.05$). All samples were studied in at least three independent experiments. Data are represented as mean ± SEM. This analysis shows protein expression downregulation for the key TFs NR3C1, HNF4A, and FOSL2 (right). Representative immunoblots in PD patients (PD) and controls (C) (left). See also source data for Fig 7.

C Immunofluorescence analysis of three DEGs showing co-localization with the DAn-specific marker tyrosine hydroxilase (TH) as illustrated in one representative sPD patient (PD-10). The transcription factors OTX2 or PAX6 showed nuclear localization, whereas the transmembrane protein DCC showed a cellular distribution. Red, OTX2, PAX6, or DCC; green, TH. Nuclei are counterstained with DAPI, shown in blue (Scale bar, 10 μm).

 © 2015 The Authors
