# Supplementary figures and images for: Aberrant epigenome in iPSC‐derived dopaminergic neurons from Parkinson's disease patients
Source: EMBO Mol Med. 2015 Oct 29;7(12):1529–46. doi: 10.15252/emmm.201505439 (PMC4693505; doi:10.15252/emmm.201505439)

Figure 7 and Figure EV3

Proteins upregulated in PD

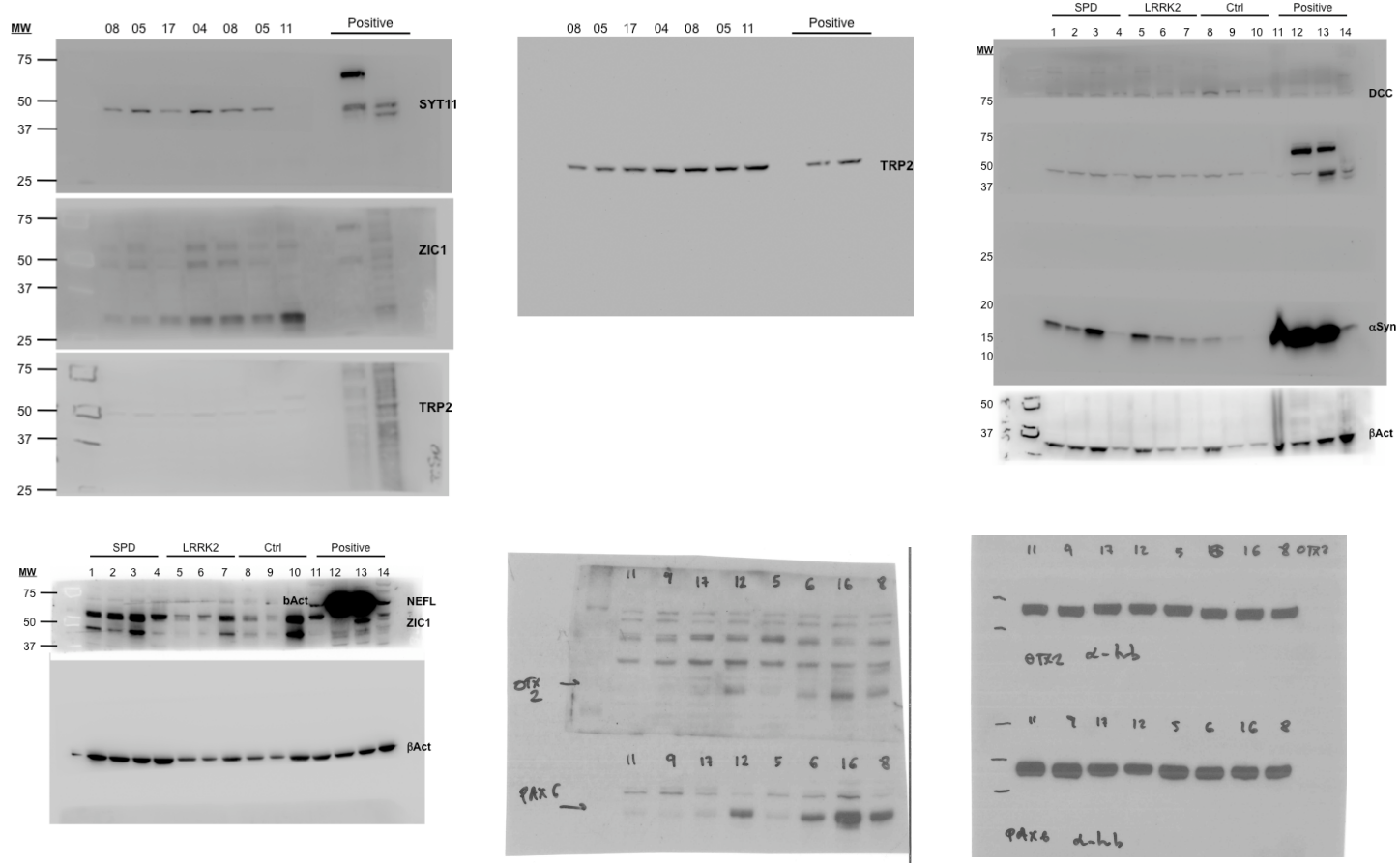

Key TFs

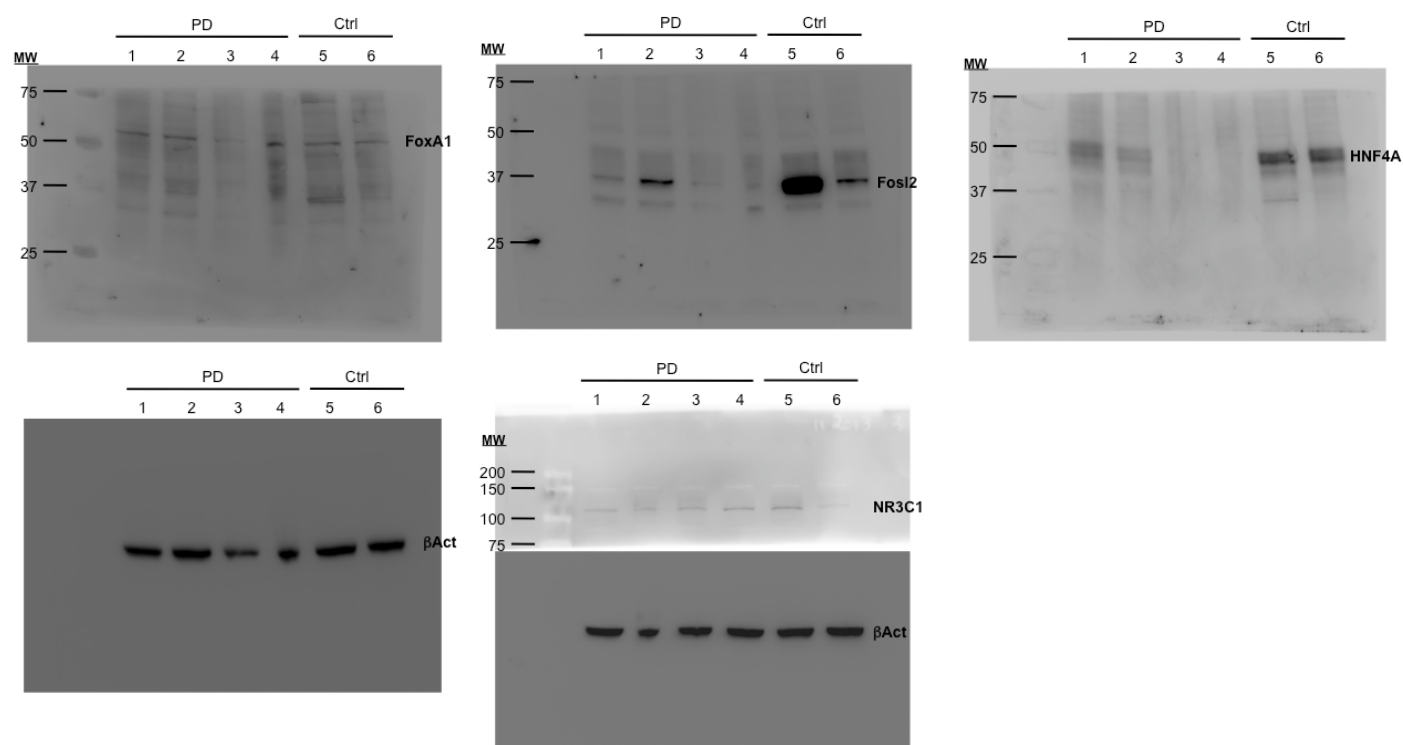

Supplement: Supplementary file 10 — Source Data for Figure 7 [file EMMM-7-1529-s009.pdf]
